# Use of hemodialysis for the treatment of intracerebral hemorrhage in patients on dabigatran with normal renal function

**DOI:** 10.5414/CNCS108253

**Published:** 2014-08-20

**Authors:** Thin Thin Maw, Brian L. Henry, Tripti Singh

**Affiliations:** 1 Division of Nephrology,; 2 Division of Cardiology, Department of Medicine, University of California, San Francisco, CA, and; 3 Renal-Electrolyte Division University of Pittsburgh School of Medicine, Pittsburgh, PA, USA

**Keywords:** dabigatran, intracerebral hemorrhage, hemodialysis

## Abstract

Dabigatran is a direct thrombin inhibitor which is approved by the Food and Drug Administration (FDA) for prevention of embolic stroke in patients with atrial fibrillation. Dabigatran has been shown to be non- inferior to warfarin in preventing stroke in patients with atrial fibrillation [1, 6]. The rate of major bleeding in patients taking dabigatran is also similar to that seen in patients on warfarin [1]. Unlike warfarin, there is currently no antidote available for reversal of anticoagulant effects of dabigatran [2, 3]. Dabigatran is excreted renally and accumulates in patients with acute kidney injury (AKI). Hemodialysis has been reported to increase clearance of dabigatran in patients with acute kidney injury and life-threatening bleeding [4, 5]. We present two cases of dabigatran-associated intracranial hemorrhage where hemodialysis was used to accelerate clearance of dabigatran from the blood in patients with normal renal function.

## Introduction 

Dabigatran is a direct thrombin inhibitor used to reduce the risk of stroke and systemic embolism in patients with atrial fibrillation and it has been shown to be as effective as warfarin [1, 6]. Dabigatran has a predictable pharmacokinetic-pharmacodynamic profile. Hence, it does not require routine monitoring which makes it easier to use for both patients and clinicians as compared to warfarin [7, 8, 9]. As the use of dabigatran increases, the hemorrhagic complications associated with its use will also increase. Dabigatran is renally excreted. As a result, patients with impaired kidney function and acute kidney injury (AKI) will develop supratherapeutic levels of dabigatran, even on the stable dose of dabigatran. There is currently no antidote available for rapid reversal of anticoagulant effects of dabigatran in actively bleeding patients [10, 11, 12]. 

The lack of a specific antidote, the drug’s reliance on renal excretion and molecular characteristics of dabigatran has led to the idea of using dialysis for removing dabigatran from patients [3]. Stangier et al. [11] showed that hemodialysis (HD) was capable of removing 62 – 68% of dabigatran from 6 ESRD patients. Other groups have documented the use of intermittent and continuous renal replacement therapy modalities and plasmapheresis in the treatment of dabigatran-associated bleeding [13, 14, 15, 16, 17]. Recently, our group published the largest case series of patients (comprising of 5 consecutive cases) with life-threatening dabigatran-associated bleeding in the setting of AKI [4]. In our case series, dabigatran concentrations decreased by 52 – 77% during intermittent HD but rebounded up to 87% within 2 hours after completion of dialysis. Initiation of continuous renal replacement therapy after intermittent HD attenuated the rebound effect. Therefore, based on our experience, we recommended the use of intermittent HD followed by continuous renal replacement therapy for the management of life-threatening bleeding in patients receiving dabigatran as long as the advantages of extracorporeal therapy have been weighed against the risks of bleeding associated with catheter insertion. 

Recently, we encountered a different clinical scenario involving patients with life-threatening dabigatran-associated hemorrhage – specifically, intracranial hemorrhage in the setting of normal dabigatran levels and normal renal function. Here we present 2 such patients. 

## Cases 

### Case 1 

A 68-year-old woman with past medical history of atrial fibrillation on dabigatran 150 mg twice a day, coronary artery disease, hypertension, and hyperlipidemia was brought to the emergency room after a motor vehicle accident (MVA). The patient took her last dose of dabigatran 7 hours prior to the MVA. Her serum creatinine on admission was 0.9 mg/dL. Her vital signs were as follows: blood pressure 127/92 mm Hg, heart rate 114/min, respiratory rate 15/min and O_2_ saturation 97% on room air. She was awake, alert, oriented to time, place, and person. She had no focal neurological deficits. The laboratory values on admission were hemoglobin 14.3 g/dL, platelet count 262,000/µL, serum creatinine 0.9 mg/dL (eGFR 66 mL/min/1.72 m^2^), and blood urea nitrogen 14 mg/dL. The coagulation parameters on admission showed an internal randomized ratio (INR) of 1.4 (0.8 – 1.2), activated partial thromboplastin time (aPTT) of 38.2 seconds (22 – 35 seconds), prothrombin time (PT) of 16.5 seconds (11 – 17 seconds), and thrombin time > 150 seconds (16 – 22 seconds). A CT head showed multi-compartmental acute intracranial hemorrhage including bilateral frontoparietal subarachnoid hemorrhage and a small left frontal lobe intraparenchymal hemorrhage. She received 1 dose of Factor VIIa (40 µg/kg) 2 hours prior to insertion of right femoral HD catheter. She received 1 session of 4 hours of HD on the night of admission for removal of dabigatran. Dialysis was performed with a high-flux dialyzer (REXEED 25) and Gambro HD machines without any anticoagulation. The dialysate flow rate was 800 mL/min and the blood flow rate was 400 mL/min. Dabigatran concentration was 123 ng/mL on admission and decreased to 57 ng/mL after 2 hours of HD and to undetectable level (< 40 ng/mL) at the end of the 4-hour HD treatment. The thrombin time was > 150 seconds on admission and remained elevated at > 150 seconds after 2 hours of HD but decreased to 60 seconds after completion of the 4-hour HD treatment. A repeat CT scan of the head performed 24 hours after admission revealed no worsening of intracranial hemorrhage. The patient was discharged home after 5 days of hospitalization without any anticoagulation. 

### Case 2 

A 65-year-old woman with past medical history of atrial fibrillation on dabigatran 75 mg twice a day, stroke, coronary artery disease, diabetes mellitus on insulin pump, hypertension, hyperlipidemia, and hypothyroidism came to the emergency room after a mechanical fall. The patient had taken her last dose of dabigatran 4 hours prior to arrival at the emergency room. Her baseline serum creatinine was 0.8 mg/dL. Her vital signs were as follows: blood pressure 134/58 mm Hg, heart rate 69/min, respiratory rate 15/min and O_2_ saturation 99% on room air. She was awake, alert, oriented to time, place, and person. She had skin abrasions on her forehead and clotted blood in her nose. She had right-sided residual weakness from her previous stroke, but no new focal neurological deficits. The laboratory values on admission were hemoglobin 13.8 g/dL, platelet count 211,000/µL, serum creatinine 0.9 mg/dL (eGFR 66 mL/min/1.72 m^2^) and blood urea nitrogen 20 mg/dL. The coagulation parameters on admission showed an INR of 1.2, aPTT of 36.5 seconds, PT of 14.7 seconds and thrombin time > 150 seconds. A CT scan of the head revealed acute left subdural hematoma with overlying scalp hematoma. She received 1 unit of platelets and 1 dose of Factor VIIa (40 µg/kg) 3 hours prior to insertion of left HD catheter. She received 1 session of 4 hours of HD on the day of admission for removal of dabigatran. Dialysis was performed with a high-flux dialyzer (REXEED 25) and Gambro HD machines without any anticoagulation. The dialysate flow rate was 800 mL/min and the blood flow rate was 400 mL/min. Dabigatran concentration was 41 ng/mL on admission and decreased to an undetectable level (< 40 ng/mL) at the end of the 4-hour HD treatment. The thrombin time decreased to 39.3 seconds after the completion of the 4-hour HD treatment. Repeat CT scan 48 hours after admission showed stable left sided subdural hematoma. The patient was discharged home after 5 days without any anticoagulation.[Table Table1]

## Discussion 

Dabigatran is a direct thrombin inhibitor approved by the Food and Drug Administration (FDA) to reduce the risk of stroke in patients with atrial fibrillation [2]. Dabigatran has been shown to be non-inferior to warfarin in preventing stroke in patients with atrial fibrillation while carrying a similar or lower bleeding risk [1, 6]. In the RE-LY trial, the rate of major bleeding was 3.36% per year in the warfarin group, as compared to 3.11% per year in the group that received 150 mg of dabigatran (relative risk, 0.93; 95% CI, 0.81 – 1.07; p = 0.31). However, the rates of intracranial bleeding were significantly higher with warfarin (0.74% per year), as compared to the 150-mg dose of dabigatran (0.30% per year, p < 0.001). This data suggests that patients on dabigatran are less likely to develop intracerebral hemorrhage compared to patients taking warfarin. But as the clinical use of dabigatran increases, the number of patients with hemorrhagic complications will also increase. 

Approximately 80% of dabigatran is excreted renally. The half-life of dabigatran is ~ 12 hours in patients with stable renal function [9]. The FDA has recommended a dabigatran dose of 150 mg twice daily in patients with a creatinine clearance (CrCl) > 30 mL/min and 75 mg twice daily with a CrCl of 15 – 30 mL/min [2, 3]. Dabigatran is contraindicated in patients with CrCl < 15 mL/min. In case 2, the patient was inappropriately on 75 mg twice daily as her baseline CrCl > 30 mL/min, but case 1 was on the correct dose of 150 mg twice daily. The dabigatran dose of 150 mg twice daily produces a steady-state peak and trough concentration of ~ 180 mg and ~ 90 mg, respectively, assuming a CrCl > 60 mL/min [18]. However, the disadvantage of dabigatran is that there is currently no rapid laboratory test available to accurately measure the anticoagulant effect of dabigatran. Also, there is currently no antidote available to reverse the anticoagulant effects of dabigatran. 

In both our cases, the patients presented with normal renal function and did not develop AKI during the hospital stay. The patient in case 1 took 150 mg of dabigatran 7 hours prior to the presentation and the patient in case 2 took a 75-mg dose 4 hours prior to the presentation. Since the half-life of dabigatran is ~ 12 hours in patients with normal renal function, the patient in case 1 will experience the anticoagulant effect of dabigatran for ~ 5 hours and the patient in case 2 for ~ 8 hours from the time of presentation. It has been suggested that the administration of activated charcoal with sorbitol may be useful in decreasing the absorption of dabigatran. However, this would work only if the patient had taken the medication within the last 2 hours [19]. Therefore, this was not an option for these patients. Also, there is currently no readily accessible laboratory marker that rapidly measures the anticoagulant effect of dabigatran. aPTT has been shown to be elevated in patients on dabigatran but at supratherapeutic levels of dabigatran, the relationship is not linear [20]. Thrombin time (TT) directly measures thrombin activity and is extremely sensitive to dabigatran [7]. In fact, TT is so sensitive that even low levels of dabigatran can prolong TT beyond the upper limits of detection [20]. This was demonstrated in case 2, where the patient had a dabigatran level of 41 ng/dL but a TT > 150 seconds. Ecarin clotting time (ECT) appears to be able to accurately quantify the degree of anticoagulation caused by dabigatran but it is not readily available for clinical use [20]. The laboratory at our institution cannot measure ECT. It is also important to note that PT and INR, although readily and rapidly available, have no role in the determination of the degree of anticoagulation caused by dabigatran [4, 20, 21]. In our institution, we are equipped to directly measure dabigatran levels by diluted thrombin time clotting assay which will correlate with its anticoagulant effect. However, it takes ~ 24 hours to get the result which is too long to wait to decide about any intervention in an emergent situation. In both of our cases, the patients had acute intracranial hemorrhages that were believed to be potentially life-threatening by the neurosurgery team directing the patient’s care. HD was used to accelerate dabigatran clearance from the intravascular space and to reverse its anticoagulant effects as no other antidote is currently available or has been documented to be superior to dialysis. 

Based on our earlier experience with patients who were experiencing life-threatening hemorrhage with AKI, we knew that HD could effectively remove dabigatran from the intravascular space [4]. For both cases, we observed that a 4-hour high-flux HD was able to decrease the pre-dialysis dabigatran concentration from 41 ng/dL and 123 ng/dL to undetectable levels (< 40 ng/dL) post-dialysis, demonstrating that HD successfully removed dabigatran from the intravascular compartment. As secondary evidence, coagulation parameters like TT and aPTT time also corrected to normal values along with reduction in dabigatran concentration after completion of HD. 

## Limitations 

HD is an invasive procedure that requires insertion of a HD catheter which is associated with risk of bleeding especially in patients with deranged coagulation profile. The risks of initiation of HD should be carefully weighed against the benefit of HD in patients with a life threatening hemorrhage. 

## Conclusion 

We suggest that HD exists as a therapeutic option for patients who have life-threatening bleeding while on dabigatran. Intracranial hemorrhage is associated with a high mortality rate, or in case of survivors, a high morbidity. We have advanced the concept that even in patients with normal renal function, the use of HD can be implemented in the event of intracranial hemorrhage to accelerate dabigatran removal. The purpose of this case report is to provide guidance to physicians in emergency department and nephrologists to consider HD as a life-saving option after conservative measures have failed for the management of dabigatran associated life threatening bleeding to accelerate the clearance of dabigatran even in patients with normal renal function. 

## Conflicts of interest 

None. 

**Table 1. Table1:** Laboratory parameters and therapeutic interventions.

	Case 1	Case 2
Vital signs		
Pulse (beats/min)	114	69
BP (mmHg)	127/92	134/58
Laboratory parameters		
Baseline SCr (0.5 – 1.4 mg/dL)	NA	0.8
Admission SCr (mg/dL)	0.9	0.9
Peak SCr (mg/dL)	1.1	0.9
Hemoglobin (11.6 – 14.6 g/dL)	14.3	13.8
Platelets (156 – 369 k/cumm)	262	211
Pre-HDaPTT (22.7 – 35.6sec)	38.2	36.5
Post-HD aPTT (sec)	33.2	26.3
Pre-HD TT (16 – 22 sec)	>150	>150
Post-HD TT (sec)	60	39.3
Pre-HD INR (0.8 – 1.2)	1.4	1.2
Post-HD INR	1.0	0.9
Pre-HD dabigatran (ng/dL)	123	41
Post-HD dabigatran (ng/dL)	< 40	<40
Therapeutic interventions		
Platelets (units)	None	1
rFactor VII (mg)	3	2.5
HD duration (hours)	4	4
QD (mL/min)	800	800
QB (mL/min)	400	400
HD catheter	Left femoral	Right femoral

BP = blood pressure; SCr = serum creatinine; HD = hemodialysis; aPTT = activated partial thromboplastin time; PT = prothrombin time; INR = international normalization ratio; TT = thrombin clotting time; rFactor VII = recombinant Factor VII; QD = dialysate flow rate; QB = blood flow rate; NA = not available.

## References

[1] Connolly SJ Ezekowitz MD Yusuf S Eikelboom J Oldgren J Parekh A Pogue J Reilly PA Themeles E Varrone J Wang S Alings M Xavier D Zhu J Diaz R Lewis BS Darius H Diener HC Joyner CD Wallentin L Dabigatran versus warfarin in patients with atrial fibrillation. N Engl J Med. 2009; 361: 1139–1151. 19717844 10.1056/NEJMoa0905561

[2] FDA Drug Safety Communication. Safety review of post-market reports of serious bleeding events with the anticoagulant Pradaxa (dabigatran etexilate mesylate), 2011. Available at: http://www.fda.gov/Drugs/DrugSafety/ucm282724.htm#sa 2011. Accessed January 21, 2013.

[3] Boehringer Ingelhein Pharmaceuticals, Inc. Pradaxa (Dabigatran Etexilate Mesylate) U.S. Full Prescribing Information. Available at: www.pradaxa.com.

[4] Singh T Maw TT Henry BL Pastor-Soler NM Unruh ML Hallows KR Nolin TD Extracorporeal therapy for dabigatran removal in the treatment of acute bleeding: a single center experience. Clin J Am Soc Nephrol. 2013; 8: 1533–1539. 23704302 10.2215/CJN.01570213 PMC3805068

[5] Knauf F Chaknos CM Berns JS Perazella MA Dabigatran and kidney disease: a bad combination. Clin J Am Soc Nephrol. 2013; 8: 1591–1597. 23868901 10.2215/CJN.01260213 PMC3805067

[6] Eikelboom JW Wallentin L Connolly SJ Ezekowitz M Healey JS Oldgren J Yang S Alings M Kaatz S Hohnloser SH Diener HC Franzosi MG Huber K Reilly P Varrone J Yusuf S Risk of bleeding with 2 doses of dabigatran compared with warfarin in older and younger patients with atrial fibrillation: an analysis of the randomized evaluation of long-term anticoagulant therapy (RE-LY) trial. Circulation. 2011; 123: 2363–2372. 21576658 10.1161/CIRCULATIONAHA.110.004747

[7] Stangier J Stähle H Rathgen K Fuhr R Pharmacokinetics and pharmacodynamics of the direct oral thrombin inhibitor dabigatran in healthy elderly subjects. Clin Pharmacokinet. 2008; 47: 47–59. 18076218 10.2165/00003088-200847010-00005

[8] Stangier J Clinical pharmacokinetics and pharmacodynamics of the oral direct thrombin inhibitor dabigatran etexilate. Clin Pharmacokinet. 2008; 47: 285–295. 18399711 10.2165/00003088-200847050-00001

[9] Stangier J Clemens A Pharmacology, pharmacokinetics, and pharmacodynamics of dabigatran etexilate, an oral direct thrombin inhibitor. Clin Appl Thromb Hemost. 2009; 15: 9S–16S. 19696042 10.1177/1076029609343004

[10] Cotton BA McCarthy JJ Holcomb JB Acutely injured patients on dabigatran. N Engl J Med. 2011; 365: 2039–2040. 22111735 10.1056/NEJMc1111095

[11] Ganetsky M Babu KM Salhanick SD Brown RS Boyer EW Dabigatran: review of pharmacology and management of bleeding complications of this novel oral anticoagulant. J Med Toxicol. 2011; 7: 281–287. 21887485 10.1007/s13181-011-0178-y PMC3550194

[12] Battinelli EM Reversal of new oral anticoagulants. Circulation. 2011; 124: 1508–1510. 21969317 10.1161/CIRCULATIONAHA.111.054510

[13] Cano EL Miyares MA Clinical challenges in a patient with dabigatran-induced fatal hemorrhage. Am J Geriatr Pharmacother. 2012; 10: 160–163. 22386824 10.1016/j.amjopharm.2012.02.004

[14] Wychowski MK Kouides PA Dabigatran-induced gastrointestinal bleeding in an elderly patient with moderate renal impairment. Ann Pharmacother. 2012; 46: e10. 22496474 10.1345/aph.1Q747

[15] Kamboj J Kottalgi M Cirra VR Shah N Kamboj R Direct thrombin inhibitors: a case indicating benefit from ‘plasmapheresis’ in toxicity: a call for establishing “guidelines” in overdose and to find an “antidote”! Am J Ther. 2012; 19: e182–e185. 23154229 10.1097/MJT.0b013e3182459a79

[16] Harinstein LM Morgan JW Russo N Treatment of dabigatran-associated bleeding: case report and review of the literature. J Pharm Pract. 2013; 26: 264–269. 23160864 10.1177/0897190012465955

[17] Chang DN Dager WE Chin AI Removal of dabigatran by hemodialysis. Am J Kidney Dis. 2013; 61: 487–489. 23219111 10.1053/j.ajkd.2012.08.047

[18] Hankey GJ Eikelboom JW Dabigatran etexilate: a new oral thrombin inhibitor. Circulation. 2011; 123: 1436–1450. 21464059 10.1161/CIRCULATIONAHA.110.004424

[19] van Ryn J Stangier J Haertter S Liesenfeld KH Wienen W Feuring M Clemens A Dabigatran etexilate--a novel, reversible, oral direct thrombin inhibitor: interpretation of coagulation assays and reversal of anticoagulant activity. Thromb Haemost. 2010; 103: 1116–1127. 20352166 10.1160/TH09-11-0758

[20] Mani H Kasper A Lindhoff-Last E Measuring the anticoagulant effects of target specific oral anticoagulants-reasons, methods and current limitations. J Thromb Thrombolysis. 2013; 36: 187–194. 23512159 10.1007/s11239-013-0907-y

[21] Douxfils J Mullier F Robert S Chatelain C Chatelain B Dogné JM Impact of dabigatran on a large panel of routine or specific coagulation assays. Laboratory recommendations for monitoring of dabigatran etexilate. Thromb Haemost. 2012; 107: 985–997. 22438031 10.1160/TH11-11-0804

